# Sustained Activation of Protein Kinase C Induces Delayed Phosphorylation and Regulates the Fate of Epidermal Growth Factor Receptor

**DOI:** 10.1371/journal.pone.0080721

**Published:** 2013-11-11

**Authors:** Mengling Liu, Jolanta Idkowiak-Baldys, Patrick L. Roddy, Aleksander Baldys, John Raymond, Christopher J. Clarke, Yusuf A. Hannun

**Affiliations:** 1 Department of Medicine and The Stony Brook Cancer Center, Stony Brook University, Stony Brook, New York, United States of America; 2 Department of Biochemistry and Molecular Biology, Medical University of South Carolina, Charleston, South Carolina, United States of America; 3 Division of Nephrology, Department of Medicine, Medical University of South Carolina, Charleston, South Carolina, United States of America; 4 Medical and Research Services, Ralph H. Johnson Veterans Affairs Medical Center, Charleston, South Carolina, United States of America; 5 Medical College of Wisconsin, Milwaukee, Wisconsin, United States of America; Tohoku University, Japan

## Abstract

It is well established that acute activation of members of the protein kinase C (PKC) family induced by activation of cellular receptors can transduce extracellular stimuli to intracellular signaling. However, the functions of sustained activation of PKC are not well studied. We have previously shown that sustained activation of classical PKC isoforms over 15-60 min induced the formation of the pericentrion, a subset of recycling endosomes that are sequestered perinuclearly in a PKC- and phospholipase D (PLD)-dependent manner. In this study, we investigated the role of this process in the phosphorylation of EGFR on threonine 654 (Thr-654) and in the regulation of intracellular trafficking and fate of epidermal growth factor receptor (EGFR). Sustained stimulation of the angiotensin II receptor induced translocation of the EGFR to the pericentrion, which in turn prevents full access of EGF to the EGFR. These effects required PKC and PLD activities, and direct stimulation of PKC with phorbol esters was sufficient to reproduce these effects. Furthermore, activation of PKC induced delayed phosphorylation of EGFR on Thr-654 that coincided with the formation of the pericentrion and which was dependent on PLD and endocytosis of EGFR. Thus, Thr-654 phosphorylation required the formation of the pericentrion. On the other hand, using a T654A mutant of EGFR, we find that the phosphorylation on Thr-654 was not required for translocation of EGFR to the pericentrion but was required for protection of EGFR from degradation in response to EGF. Taken together, these results demonstrate a novel role for the pericentrion in the regulation of EGFR phosphorylation, which in turn is important for the fates of EGFR.

## Introduction

 Protein kinase C (PKC) is a family of enzymes implicated in numerous cellular processes including proliferation, migration and cell survival. Currently, there are eleven known PKC isoforms grouped into 3 subfamilies according to their domain structure and activation. Classical PKCs (cPKCs; α, βI, βII and γ) have functional C1 and C2 domains, and are activated by phosphatidylserine (PS), diacylglycerol (DAG) and calcium[1]. The novel PKCs (nPKCs;δ, ε, η and θ) are PS- and DAG-dependent but are calcium-independent as they have truncated C2 domains[1]. Differing from both cPKCs and nPKCs, the atypical PKCs (ι, λ, ζ) have truncated C1 domains and no C2 domain [2] and are also independent of DAG activation. Notably, tumor promoting phorbol esters, such as 4β-phorbol-12-myristate-13-acetate (PMA), were found to directly activate both cPKC and nPKC isoforms by mimicking DAG [3]. In the classical paradigm, cPKCs are acutely activated upon agonist binding to tyrosine kinase or G-protein coupled receptors (GPCR) such as the angiotensin II type 1A receptor, which results in stimulation of phospholipase C (PLC) isoforms. PLC subsequently hydrolyzes phosphatidylinositol 4,5 bisphosphate to produce inositol 1,4,5- triphosphate (IP3), which releases calcium from intracellular stores, and DAG [4]. This results in translocation of cPKCs from the cytosol to the plasma membrane within 60 seconds [5], bringing it in close proximity to its substrates. 

 In addition to the above well-established paradigm of acute activation and translocation of cPKCs, it was previously reported by our group that sustained activation of cPKCs, both by PMA or by activation of GPCRs such as the serotonin receptor, resulted in internalization of cPKCs from the plasma membrane and their translocation to a perinuclear compartment involving recycling endosomes that become sequestered around pre-existing Rab11 endosomes [6,7]. Additional results revealed that this is a dynamic compartment, requiring both PKC and PLD activities and dependent on clathrin-mediated endocytosis [8]. We termed these sequestered PKC- and PLD-dependent endosomes the pericentrion. Furthermore, formation of the pericentrion did not occur at temperatures below 32 °C, which distinguished the process from general endocytosis [9]. Functionally, formation of the pericentrion also induced sequestration of recycling molecules such as transferrin, some membrane receptors (e.g. 5-HT receptor) and membrane lipids (ganglioside GM_1_) [7]. Besides sequestration of different molecules, additional studies have shown that formation of the pericentrion also coincided with the phosphorylation of a subgroup of PKC substrates including Rab11, S6 kinase and transferrin receptor [10].. 

 The epidermal growth factor receptor (EGFR) is one of the best-studied tyrosine kinase receptors. EGFR can be activated by numerous ligands including neuregulins [11] but, to date, transforming growth factor-α and epidermal growth factor (EGF) are the best studied. The binding of ligands to the extracellular domain of EGFR induces autophosphorylation on several tyrosine residues of the receptor with each site mediating specific functions of EGFR regulation [12-16]. For example, phosphorylation at tyrosine 1045 (Tyr-1045) is reported to play a key role in endocytosis and translocation of the receptor to the lysosome for degradation [17-19] and autophosphorylation at tyrosine 1068 (Tyr-1068) is a major event for EGFR activation [20]. In addition to autophosphorylation, it is also known that PKCs can phosphorylate EGFR on Thr-654, and this may protect the EGFR from degradation in the lysosome [18]. In a recent study, we showed that sustained treatment with serotonin (5-HT) led to sequestration of EGFR in the perinuclear region, and this process was dependent on cPKC and PLD activities, indicative of its localization in the pericentrion [7]. Consequently, it became of great interest to determine the mechanistic relationship between translocation to the pericentrion, phosphorylation of EGFR on Thr-654, and diversion of the EGFR into the recycling pathway.

 Here, using HEK293 cells as a model system, we provide evidence that the phosphorylation of EGFR on Thr-654 is a delayed process that requires formation of the pericentrion. Furthermore, we show that PMA induces sequestration of EGFR from the plasma membrane to the perinuclear recycling endosome in a cPKC- and PLD-dependent manner and that this sequesters EGFR from EGF binding. Notably, the protection of EGFR from EGF binding required the phosphorylation of EGFR on Thr-654. Collectively, these results identify a novel role for the pericentrion in the regulation of EGFR phosphorylation and intracellular trafficking.

## Materials and Methods

### Materials

Minimal essential medium (MEM) was from Invitrogen. The HEK293 cell line was purchased from American Type Culture Collection. HEK293 cells with stable expression of AT1AR-GFP （angiotensin II type 1A receptor) that were previously characterized [21] were gifts from Dr. Thomas A. Morinelli (Medical University of South Carolina, Charleston, SC). 4-Phorbol 12-myristate-13-acetate (PMA), GÖ6976 and Bisindolylmaleimide I (Bis) were purchased from Calbiochem. Anti EGFR, and Phospho-Thr-654 EGFR antibodies were from Upstate Biotechnology (Lake Placid, NY). Phospho-Tyr-1045 EGFR and phospho-Tyr-1068 EGFR antibodies were from Cell Signaling. Na+K+ATPase antibody was from Abcam. β-Actin antibody was from Sigma. Other antibodies were from Santa Cruz. Alexa Fluor 555 secondary antibody was from Invitrogen. DRAQ5 was from Biostatus Limited. FIPI was a gift from Dr. Michael Frohman (Stony Brook University, School of Medicine). Serotonin, epidermal growth factor, angiotensin, and all other chemicals were from Sigma.

### Cell Culture

HEK293 cells were grown in MEM supplemented with 10% (v/v) fetal bovine serum. The HEK293 cells with stable expression of AT_1A_R-GFP were grown in MEM containing 10% (v/v) fetal bovine serum (FBS) and 700 μg/ml Geneticin. All cells were grown in a 5% CO_2_ incubator at 37°C. 

### Plasmids

All plasmids were made by standard protocols. HA-tagged mutants of PLD1 (K898R) and PLD2 (K758R) were gifts from Guangwei Du (Stony Brook University, New York, NY). Human EGFR and the mutant T654A EGFR were subcloned into pEGFP-N1 vector by polymerase chain reaction (PCR). These constructs were then used to perform cell studies. The EGFR PCR products were generated with a 5’-AAAAAAAACCCAAGCTTGCGATGCGACCCTCCGGGACGGCCG GG primer containing a HindIII site and a 3’-TCGGGGTACCTTTGCTCCAATAAATTCACTGC TTTG primer that was minus the stop codon sequence and containing a KpnI site. The amplified EGFR products were then subcloned into the pEGFP-N1 vector and maxi-prepped. The vectors were then sequenced for confirmation.

### Transient Transfection, Indirect Immunofluorescence, and Confocal Microscopy

Cells were plated on 35-mm confocal dishes (MatTek) at a density 3 to 5 ×10^5^ cells/dish. After 24 hours, Lipofectamine 2000 (Invitrogen) was used for transient transfection following the manufacturer’s recommendation. Transfected cells were grown in normal medium with 10% FBS for 24 hours and then starved with medium with 0.1% bovine serum albumin (BSA) for 5 hours, followed by treatments. Cells expressing green fluorescent protein (GFP) were fixed with 3.7% formaldehyde for 10 minutes and then analyzed by confocal microscopy. For indirect immunofluorescence, the procedure was the same as described before [7]. All images were taken by LSM 510 Meta from Zeiss (ZESS 510) and pictures are representative of three fields examined from three independent experiments. 

### Immunoblotting

Protein samples were boiled for 15 min in LDS sample buffer (Invitrogen, NuPAGE®) and separated by 4-20% polyacrylamide gel (BioRad, Criterion Tris-HCl Gel). Proteins were transferred to Nitrocellulose membranes (BioRad) and the membranes were blocked in PBST with 5% nonfat dried milk for 1 hour, followed by washing with PBST and then further incubation with primary antibody overnight at 4 °C. All primary antibodies were diluted to 1: 1000 in PBST with 2% BSA. On the following day, the blots were washed with PBST and incubated with secondary antibody in PBST with 5% nonfat dried milk for 1 hour. After washing, proteins were detected by using enhanced chemiluminescence reagent (Pierce). 

### Statistics

Statistical significance was calculated with student’s t-test or by two-way ANOVA with Bonferroni Post test where appropriate. A p-level of below 0.05 was considered to be statistically significant. 

## Results

### Sustained AT-II treatment induces co-sequestration of AT_1A_R and EGFR in the pericentrion and protects EGFR from EGF-induced degradation

**Figure 1:**
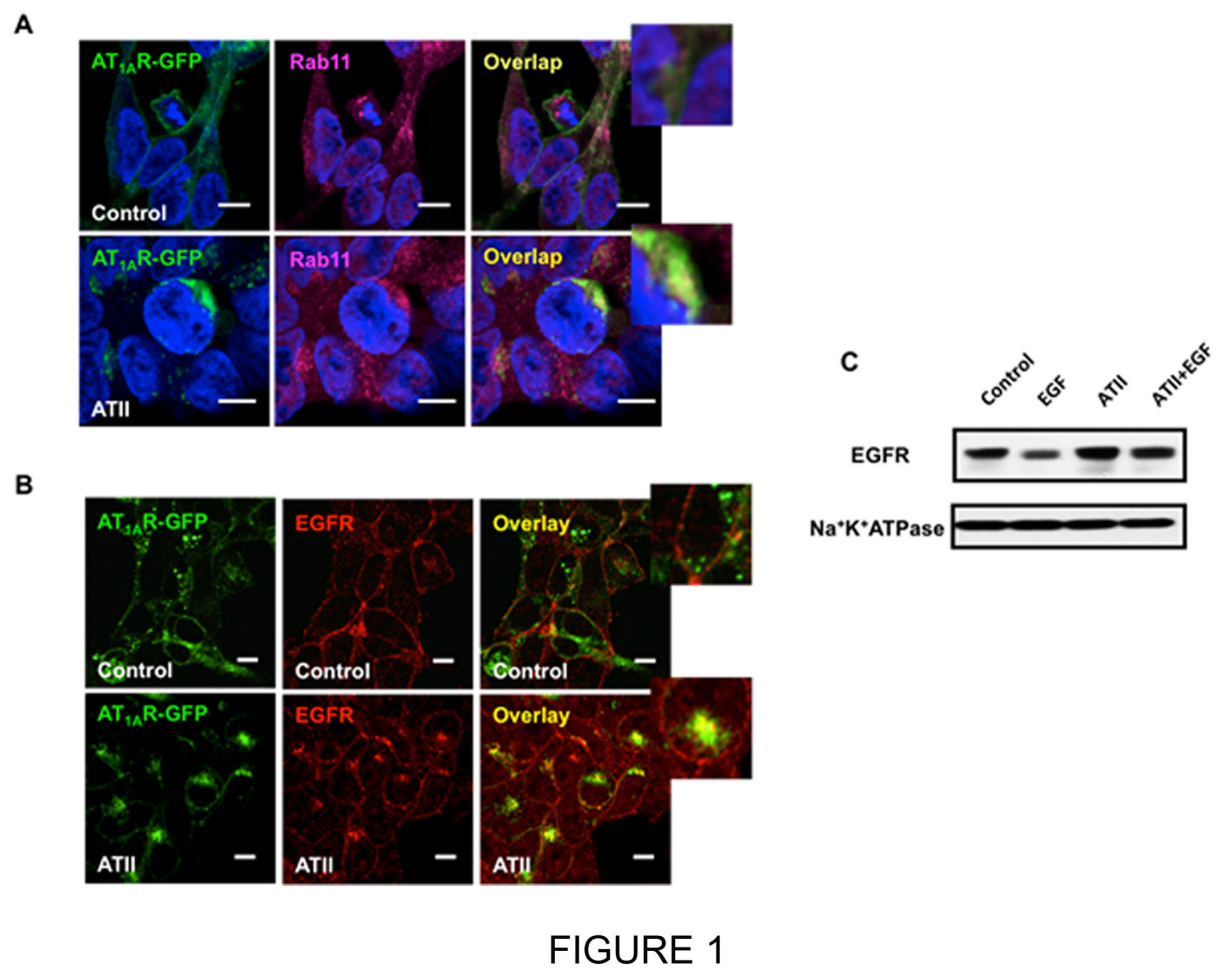
Effects of ATII on localization and fate of EGFR. A, HEK293 cells stably transfected with AT_1A_R (green) were serum starved for 5 hours followed by 100nM ATII or vehicle for 1 hour. After fixation and permeabilization, location of AT_1A_R (green) and endogenous Rab11 (red) were determined by immunofluorescence, and cells were analyzed by confocal microscopy (ZEISS 510). B, HEK293 cells stably transfected with AT_1A_R (green) were serum starved for 5 hours followed by 100nM ATII or vehicle for 1 hour. After fixation and permeabilization, endogenous EGFR (red) was determined by immunofluorescence, and cells were analyzed by confocal microscopy (ZEISS 510). C, HEK293 cells stably transfected with AT_1A_R were serum starved for 5 hours and treated with 2ng/ml of EGF for 3 hour with or without 1 hour pretreated with 100nM ATII. Protein level of EGFR was determined by Western Blotting. Blots were stripped and reprobed for Na^+^K^+^ATPase to normalize for loading. These results are representative of three independent experiments. Pictures are representative of at least three experiments.

 In our previous study, we reported that sustained stimulation with serotonin (5-HT) led to co-sequestration of the 5-HT receptor and EGFR to the pericentrion [7]. To determine if this effect extends to other GPCRs, the effects of AT-II on AT_1A_R and EGFR were examined. Utilizing HEK cells stably expressing AT_1A_R as a model system, the effects of AT-II on receptor localization were initially determined. As can be seen, AT_1A_R predominantly localized on the plasma membrane in control cells, but following prolonged AT-II treatment (60 min), the majority of AT_1A_R had translocated to a perinuclear region where it partly co-localized with Rab11, a marker of the perinuclear recycling compartment (Figure 1A). Next, the effects of AT-II on localization of both AT-II and EGFR were assessed. Again, most AT_1A_R and EGFR localized on the plasma membrane in control cells that were serum starved. As seen above, AT-II treatment induced translocation of the AT_1A_R to the pericentrion. Importantly, AT-II induced translocation of both receptors showing significant co-localization in the perinuclear compartment (Figure 1B). 

 To determine if this sequestration in the pericentrion had effects on the fate of EGFR, the effects of EGF on EGFR with pretreatment with AT-II were determined. As can be seen, EGF treatment induced a significant loss of the EGFR protein; however, with the pretreatment of AT-II, the EGF didn’t induce the loss of EGFR protein (Figure 1C). Collectively, these results show that AT-II induces translocation of both the AT_1A_R and the heterologous receptor, EGFR to the pericentrion with the functional consequence of inhibiting the loss of EGFR induced by EGF.

### AT-II-induced sequestration of the EGFR and inhibition of EGF-induced loss require the formation of the pericentrion

**Figure 2:**
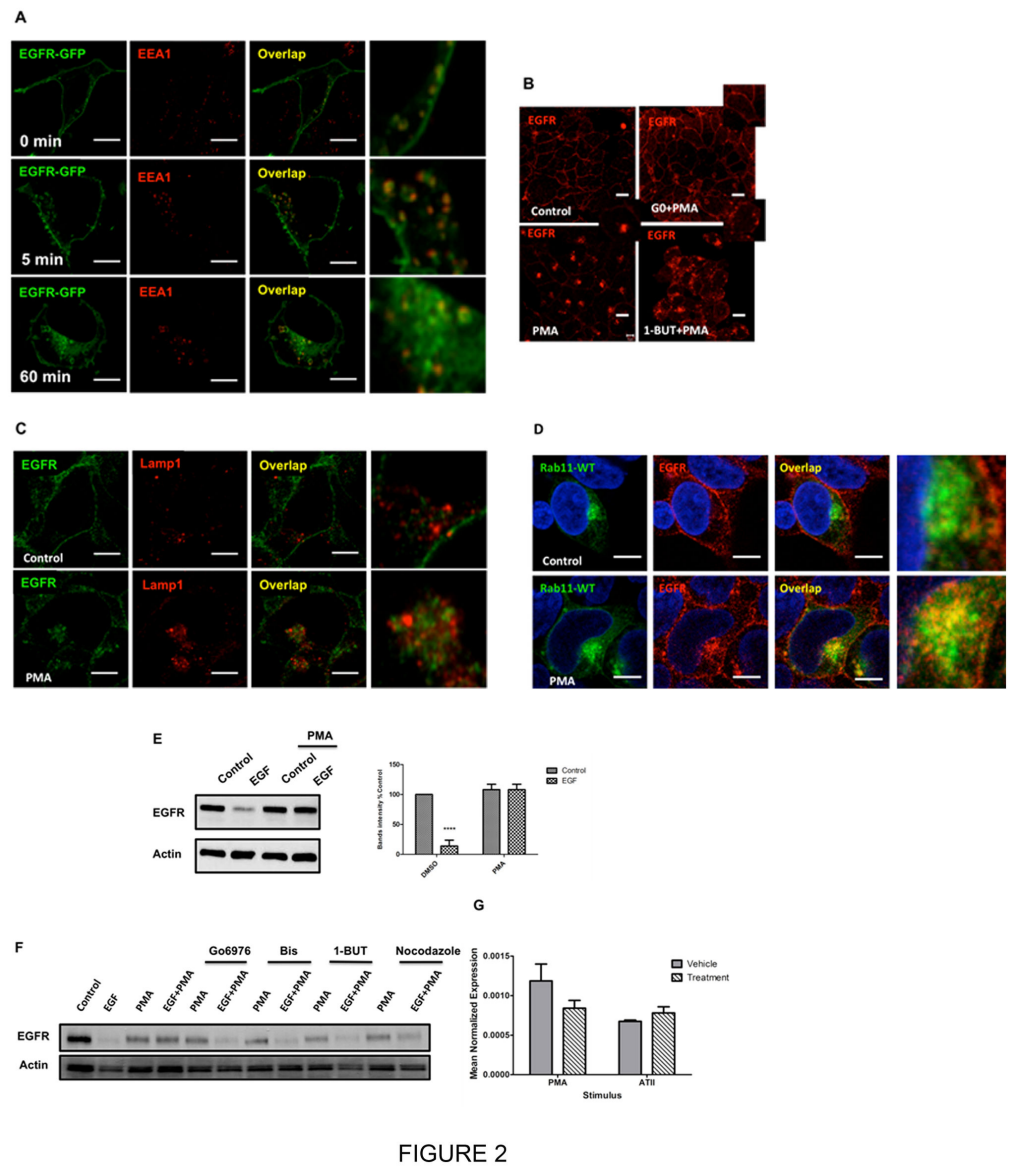
ATII-induced sequestration and protection of EGFR loss require the pericentrion. A, HEK293 cells were transfected with WT-EGFR-GFP. After 24 hours, cells were starved for 5 hours and then treated with 100nM PMA for 5 or 60 min. After fixation and permeabilization, location of EGFR (green) and endogenous EEA1 (red) were determined by immunofluorescence, and cells were analyzed by confocal microscopy (Leica TSC SP8). B, HEK293 cells were starved for 5 hours and then pretreated with Gö6976, 1-butanol or vehicle followed by 100nM PMA or vehicle for 1 hour. After fixation and permeabilization, endogenous EGFR (red) was determined by immunofluorescence, and cells were analyzed by confocal microscopy (ZEISS 510). C, HEK293 cells were starved for 5 hours and then treated with 100nM PMA or vehicle for 1 hour. After fixation and permeabilization, endogenous EGFR (green) and Lamp1 (red) were determined by immunofluorescence, and cells were analyzed by confocal microscopy (Leica TSC SP8). D, HEK293 cells were transfected with Rab11-GFP. After 24 hours, cells were starved for 5 hours and then treated with 100nM PMA or vehicle for 1 hour. After fixation and permeabilization, location of Rab11 (green) and endogenous EGFR (red) were determined by immunofluorescence, and cells were analyzed by confocal microscopy (Leica TSC SP8). E, HEK293 cells starved for 5 hours and then treated with 100nM PMA or vehicle for 1 hour following with 5ng/ml EGF or vehicle for 3 hours. Protein levels of EGFR and actin were determined by western blotting. F, HEK293 cells starved for 5 hours and then were pretreated with Gö6976, Bis, 1-butanol or vehicle followed with 100nM PMA for 1 hour and then treated with 5ng/ml EGF or vehicle for 3 hours. Protein levels of EGFR and actin were determined by western blotting. G, HEK293 cells or HEK293 cells stably overexpressing AT_1A_R were starved for 5 hours and then treated with vehicle, 100nM PMA or 100nM ATII for 1 hour. After treatments, cells were collected immediately and the mRNA level of EGFR were determined by real-time PCR. Pictures are representative of at least three experiments. E: **** *P*<0.0001 compared to control, two-way ANOVA.

 The above results show that AT-II induces sequestration of EGFR in the pericentrion and inhibits it from the loss induced by EGF. Therefore, it became important to determine if the pericentrion is necessary for the inhibition. To explore this, PMA was used as a primary inducer of the pericentrion, as reported both by our laboratory and that of Exton (8,19). For this, two different time points of PMA stimulation were used - 5 min, associated with PKC localization to the PM and 60 min, associated with formation of the pericentrion . As before, the majority of EGFR basally localized at the plasma membrane. However, after 5 min of PMA treatment, some EGFR had begun to internalize and partially co-localized with early endosome marker EEA1. In contrast, by 60 min of PMA treatment, the majority of EGFR had translocated to the perinuclear region and did not significantly co-localize with EEA1, suggesting that the internalized EGFR has left the early endosomes (Figure 2A). 

 We have previously characterized the pericentrion as a dynamic compartment requiring PKC and PLD activity for its formation (8) and therefore, the roles of PKC and PLD in regulating the cellular fate of EGFR were examined. As can be seen, pre-treatment with the cPKC inhibitor Gö 6976 (1h) prevented PMA-induced translocation of EGFR to the pericentrion (Figure 2B). Likewise, following inhibition of PLD by pretreatment with 0.4% 1-butanol, EGFR was not sequestered in the perinuclear compartment, but was present in large vesicles dispersed throughout the cell (Figure 2B). Thus, both cPKC and PLD activities are required for EGFR sequestration induced by PMA. To further confirm that EGFR translocates to the pericentrion with sustained activation of PKC, we performed co-localization studies of EGFR with Rab11, previously identified as a marker of the pericentrion (Figure 2D). As can be seen, sustained PMA stimulation induced a strong co-localization of EGFR with Rab11 but, importantly, this was completely distinct from the lysosomes (Figure 2C, D). Indeed, although PMA induced clustering of EGFR and LAMP1 within the same area, there was no obvious overlap in signal (Figure 2C). Taken together, this suggests that PMA induces trafficking of the EGFR to the pericentrion (the PKC-containing subset of Rab11 positive recycling endosomes) but does not target EGFR to the lysosomes. 

 As PMA induced EGFR localization to the pericentrion, this suggested that it might also protect EGFR from degradation induced by EGF. To assess this, cells were pre-treated with PMA prior to stimulation with EGF. As can be seen, PMA treatment did indeed lead to inhibition of the loss of EGFR induced by EGF. (Figure 2E) Importantly, both the Gö 6976 (cPKC inhibitor) and bisindolylalemide (cPKC and nPKC inhibitor) prevented the ability of PMA to induce the inhibition (Figure 2F). Similarly, inhibition of PLD with 1-butanol and distruption of microtubules with nocodazole, which have been shown to disrupt the pericentrion [22], also prevented PMA-induced protection of the EGFR (Figure 2F). To confirm that PMA and AT-II were regulating EGFR at the protein level, and not through transcriptional mechanisms, the effects of PMA and AT-II on EGFR mRNA levels was assessed by qRT-PCR. As shown in Figure 2G, PMA had a small but statistically insignificant decrease in EGFR mRNA whereas AT-II treatment had no major effect on EGFR mRNA. This confirms that sustained PKC activation regulates EGFR at the protein level rather than through transcriptional regulation.

### AT-II induces alterations in EGFR phosphorylation

**Figure 3:**
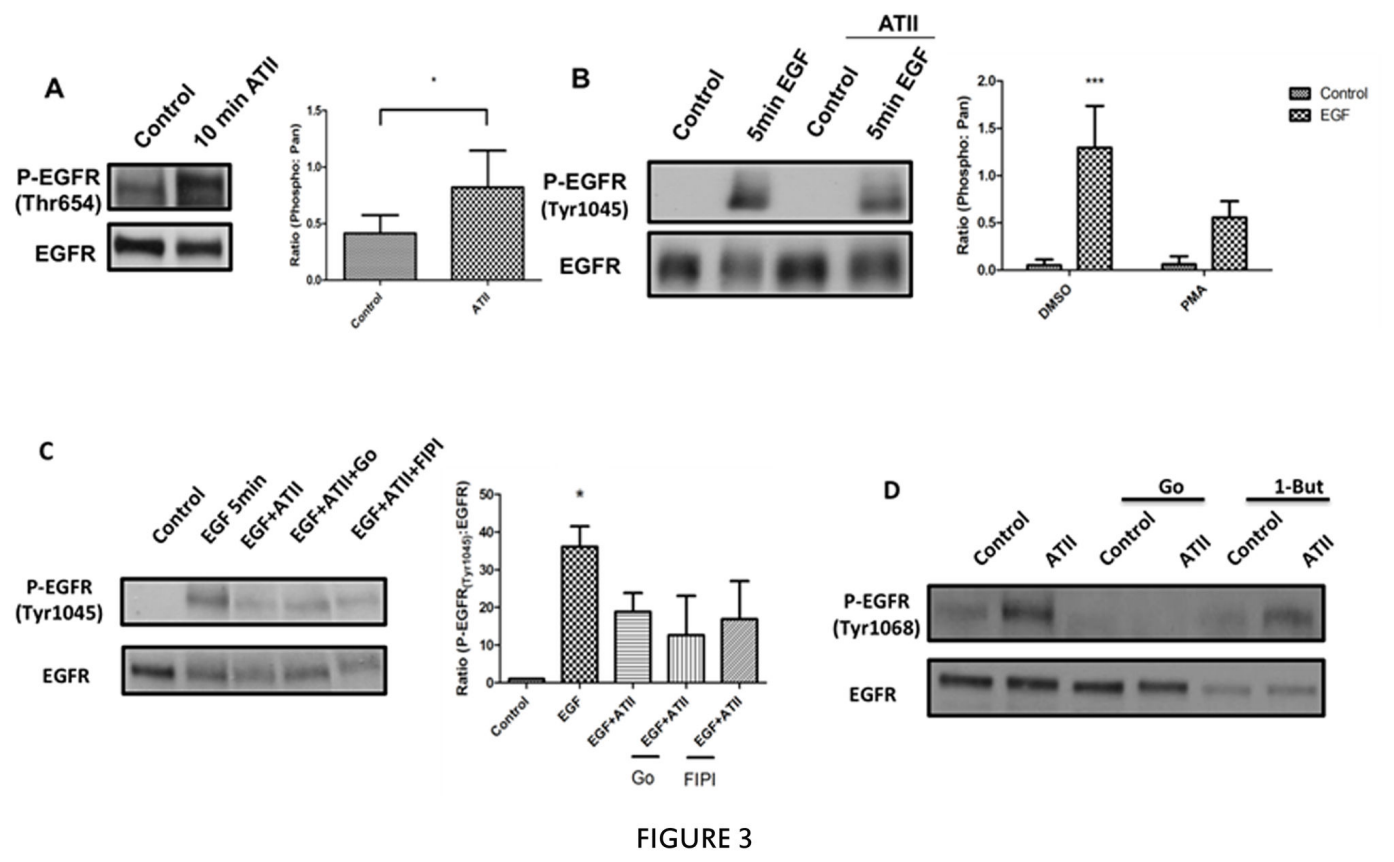
Effects of ATII on phosphorylation of EGFR. A, HEK293 cells stably transfected with AT_1A_R were serum starved for 5 hours followed by 100nM ATII for 10 min. Phosphorylation of EGFR on Thr-654 (P-Thr654) was determined by western blotting. Blots were stripped and reprobed for total EGFR to normalize for loading. B, HEK293 cells stably transfected with AT_1A_R were serum starved for 5 hours and treated with 5ng/ml of EGF for 5 min with or without 1 hour pretreated with 100nM ATII. Phosphorylation of EGFR on Tyrosine 1045 (P-Tyr1045) was determined by western blotting. The blots were stripped and reprobed for total EGFR to normalize for loading. C, HEK293 cells stably transfected with AT_1A_R were serum starved for 5hours and then pretreated with Gö6976, FIPI or vehicle followed with 100nM ATII or vehicle for 1 hour and then treated with 5ng/ml EGF for 5 min. P-Tyr1045 and EGFR were determined by western blotting. D, HEK293 cells stably transfected with AT_1A_R were serum starved for 5hours and then pretreated with Gö6976, 1-butanol or vehicle followed with 100nM ATII or vehicle for 5min, P-Tyr1068 and EGFR were determined by western blotting. For all figures, * p <0.05, ***p <0.001 from at least three independent experiments.

 Previous research has reported that phosphorylation of EGFR at various residues is important for regulating its trafficking. Indeed, PMA-induced phosphorylation of EGFR on Thr-654 was reported to change its fate from the lysosomes to the recycling endosomes [18]. In contrast, phosphorylation of EGFR on tyrosine 1045 (Tyr-1045) was found to be necessary for binding to c-Cbl, receptor ubiquitination, and degradation [23]. As the pericentrion is a subset of recycling endosomes and is required for the AT-II-induced inhibition, it became important to determine the role of these phosphorylation sites in this process. Initially, the effects of AT-II on phosphorylation of EGFR at Thr-654 and Tyr-1045 were determined. As can be seen (Figure 3A), there was a basal phosphorylation of Thr-654 in unstimulated cells that was strongly increased by AT-II treatment. In contrast, basal phosphorylation of EGFR at Tyr-1045 was minimal and was sharply increased by EGF treatment, consistent with induction of EGFR degradation. However, while AT-II had no effect on basal Tyr-1045 phosphorylation, pretreatment of cells with AT-II partially inhibited the phosphorylation of Tyr-1045 induced by EGF (Figure 3B). As Tyr-1045 phosphorylation is important for EGFR degradation, this could account for the observed effect of AT-II on the inhibition of the loss of EGFR. If this were the case and, given results suggesting that EGFR sequestration in the pericentrion is required to prevent EGFR degradation (Figure 2E), we reasoned that the effect of AT-II on Tyr-1045 would be sensitive to inhibitors of pericentrion formation; consequently, cells were treated with inhibitors of cPKC (Gö6976) and PLD (FIPI) (Figure 3C). The results showed that inhibition of either PKC or PLD [24,25] did not prevent the effects of AT-II on Tyr-1045 phosphorylation, thereby suggesting that Tyr-1045 phosphorylation occurs independently of the pericentrion.

Finally, it has also been shown that GPCR ligands can stimulate the cleavage of pro-forms of high efficacy EGFR ligands that activate the receptor but induce lower levels of EGFR degradation [26]. To assess this possibility, the effect of AT-II on phosphorylation of the EGFR residue Tyr-1068, a major autophosphorylation site on EGFR that functions as a binding site for the Grb2 adaptor protein and a marker of receptor activation were assessed. Moreover, to determine if the pericentrion is involved in any effects, the dependency of this process on PKC and PLD activities was determined (Figure 3D). The results showed that, as with Thr-654, AT-II increased the Tyr-1068 phosphorylation consistent with EGFR transactivation; however, while inhibition of cPKCs with Gö6976 inhibited this phosphorylation, PLD inhibition with 1-Butanol had no effect (Figure 3D). This indicates that transactivation of EGFR by AT-II is PKC- but not PLD- dependent. Collectively, these results suggest that, unlike AT-II-induced protection, phosphorylation of the EGFR at both Tyr-1045 and Tyr-1068 are independent of PLD and, by extension, the pericentrion.

### The pericentrion is required for Thr-654 phosphorylation on the EGFR

**Figure 4:**
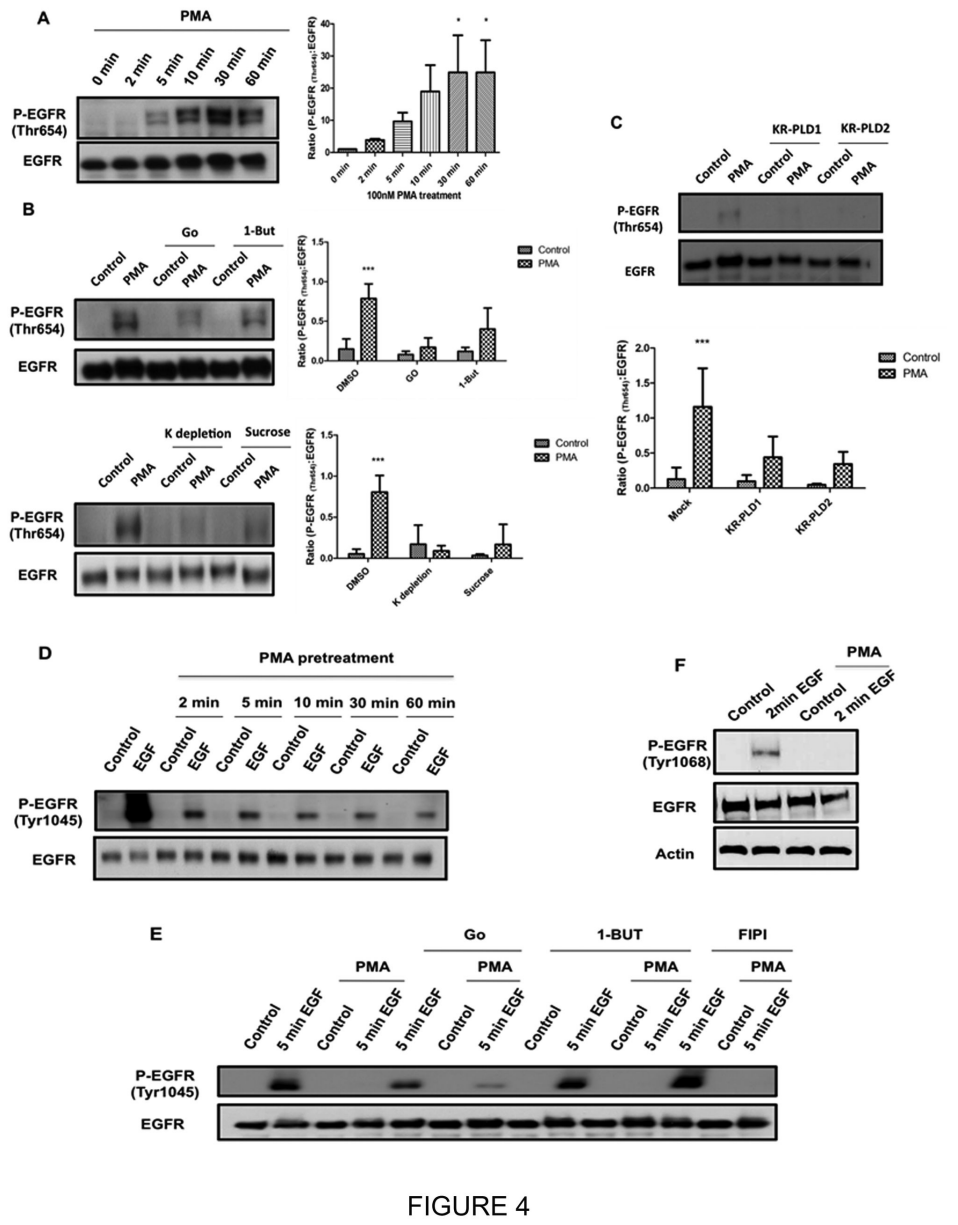
Effects of PMA on phosphorylation of EGFR and the role of the pericentrion. A, HEK293 cells were serum starved for 5 hours followed by 100nM PMA for 2 min, 5 min, 10 min, 30 min or 60 min. Phospho-Thr654 and total EGFR were determined as described above. B. HEK293 cells were starved for 5 hours and then pretreated with vehicle, Gö6976, 1-butanol, depleted of potassium (K-), or preincubated 400mM sucrose followed by 1-hour 100nM PMA treatment. The procedure for potassium-depletion is as described previously (30). Levels of P-Thr654 and total EGFR were determined as described. C, HEK293 cells were transfected with dominate negative constructs of PLD1 or PLD2. After 24 hours post-transfection, cells were starved for 5 hours and then treated with 100nM PMA for 1 hour. Levels of P-Thr654 and total EGFR were determined as shown before. D, HEK293 cells were starved for 5 hours and then pretreated with 100nM PMA for the indicated time followed by 5min 5ng/ml EGF treatment. Phosphorylation of EGFR on Tyrosine 1045 (P-Tyr1045) and total EGFR were determined as described. E, HEK293 cells were starved for 5 hours and then pretreated with Gö6976, 1-butanol, FIPI, or vehicle followed with 100nM PMA or vehicle for 1 hour and then treated with 5ng/ml EGF or vehicle for 5 min. P-Tyr1045 and EGFR were determined by western blotting. F, HEK293 cells were starved for 5 hours and then pretreated with vehicle or 100nM for 1 hour followed by treatment with 10ng/ml EGF for 2 min. Phospho-Tyr1068 and EGFR were determined by western blotting. For all figures, * p <0.05, ***p <0.001 from at least three independent experiments.

 Thus far, the results demonstrated a role for the pericentrion in the AT-II induced protection of EGFR, and suggested that this is not through effects on Tyr-1045 phosphorylation. As previous results have reported that phosphorylation of EGFR at Thr-654 is PKC-mediated and is important for regulating EGFR fate [18], it became important to determine the role of the pericentrion in this process. Initially, the effects of PMA on Thr-654 phosphorylation were determined. As can be seen (Figure 4A), PMA initiated phosphorylation of Thr-654 at 10 min with levels being sustained for the course of the experiment (Figure 4A). Importantly, this time course of phosphorylation is significantly later than initial events mediated by PKC at the plasma membrane (which occurs within 30-60 seconds) and is more coincident with formation of the pericentrion.

 To further implicate the pericentrion in regulating Thr-654 phosphorylation, we reasoned that disrupting pericentrion formation would prevent the effects of PMA on Thr-654. As noted above, we have previously established the pericentrion as cPKC and PLD-dependent [6,8,9].. Consistent with this, inhibition of cPKC (Gö6976) and PLD (1-Butanol) prevented PMA phosphorylation of Thr-654 (Figure 4B). We have also found that clathrin-dependent endocytosis is required for PMA induction of the pericentrion. Accordingly, to inhibit clathrin-dependent endocytosis, cells were either depleted of potassium or preincubated in the presence of high concentration of sucrose [27]. As with cPKC and PLD inhibitors, both these treatments inhibited PMA-induced Thr-654 phosphorylation (Figure 4B). This demonstrates that internalization of EGFR and PKC is required for Thr-654 phosphorylation in response to PMA. In order to corroborate the effects of PLD inhibition and to define the role of individual PLD isoforms in this phosphorylation, HEK293 cells were transfected with dominant negative constructs of PLD1 and PLD2 – both of which have been shown to inhibit pericentrion formation [8]. The results show that, as with the pericentrion, both isoforms are required for Thr-654 phosphorylation induced by PMA (Figure 4C). 

Data above with AT-II suggest that, unlike Thr-654, regulation of EGFR phosphorylation on Tyr-1045 is independent of the pericentrion. To further confirm this, the effects of PMA on Tyr-1045 phosphorylation were determined. Strikingly, effects observed were very rapid with PMA inhibiting EGF-induced Tyr-1045 phosphorylation with as early as 2 min of pretreatment (Figure 4D). This is in sharp contrast to the delayed effect on Thr-654, and suggests that this inhibitory effect of PMA precedes formation of the pericentrion. To confirm this, the involvement of cPKCs and PLD in the effects of PMA on Tyr-1045 was studied. As with AT-II, there was some recovery, albeit modest, of Tyr-1045 phosphorylation following inhibition of cPKC; importantly, no effect of the PLD inhibitors 1-But or FIPI on Tyr-1045 phosphorylation levels was observed (Figure 4E), confirming that PMA effects on Tyr-1045 do not require PLD or the pericentrion, and suggest that perhaps nPKCs and not cPKCs mediate this effect of PMA on Tyr-1045 phosphorylation. Collectively, these data show a requirement for PLD in phosphorylation of a cPKC substrate and demonstrate that the pericentrion is essential for PKC-mediated phosphorylation of Thr-654 but not Tyr-1045, and define compartment-specific phosphorylation events for EGFR. 

Finally, the data above suggest that AT-II is transactivating the EGFR, as evidenced by increase Tyr-1068 phosphorylation. However, it is unclear if transactivation of the receptor is necessary for sequestration in the pericentrion. To analyze this, the effects of PMA stimulation on Tyr-1068 phosphorylation were examined. As shown in Figure 4F, acute EGF induced a strong increase in Tyr-1068 phosphorylation consistent with EGFR activation. However, PMA had no significant effect suggesting that PMA does not induce transactivation. Furthermore, pretreatment with PMA completely blunted EGF-induced phosphorylation of Tyr-1068, indicating that sequestration of the EGFR in the pericentrion renders the receptor inaccessible to EGF.

### Phosphorylation of Thr-654 is required for PMA-induced protection of EGFR away from EGF but not sequestration of the EGFR

**Figure 5:**
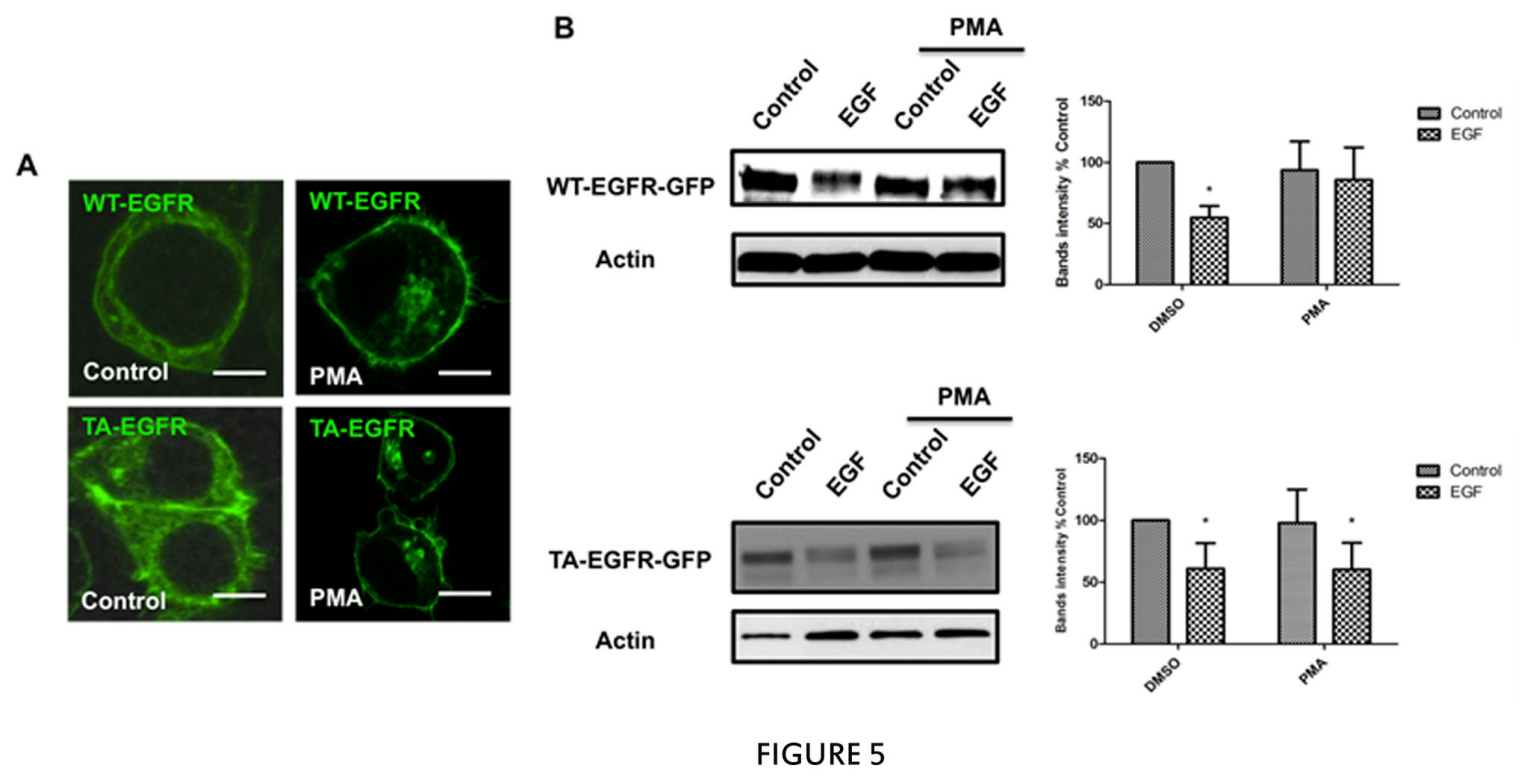
Effects of PMA on EGFR T654A mutant. A, HEK293 cells were transfected with WT-EGFR-GFP or TA-EGFR-GFP. 24 hours after transfection, cells were starved for 5 hours and then treated with 100nM PMA or vehicle for 1 hour. After fixation, cells were analyzed by confocal microscopy (ZEISS 510). B, HEK293 cells were transfected with WT-EGFR-GFP or TA-EGFR-GFP. 24 hours after transfection, cells were starved for 5 hours and then pretreated with 100nM PMA or vehicle for 1 hour followed by 5ng/ml EGF for 3 hours. The levels of EGFR, EGFR-GFP and actin were determined by western blotting. For all figures, * p <0.05, from at least three independent experiments.

**Figure 6:**
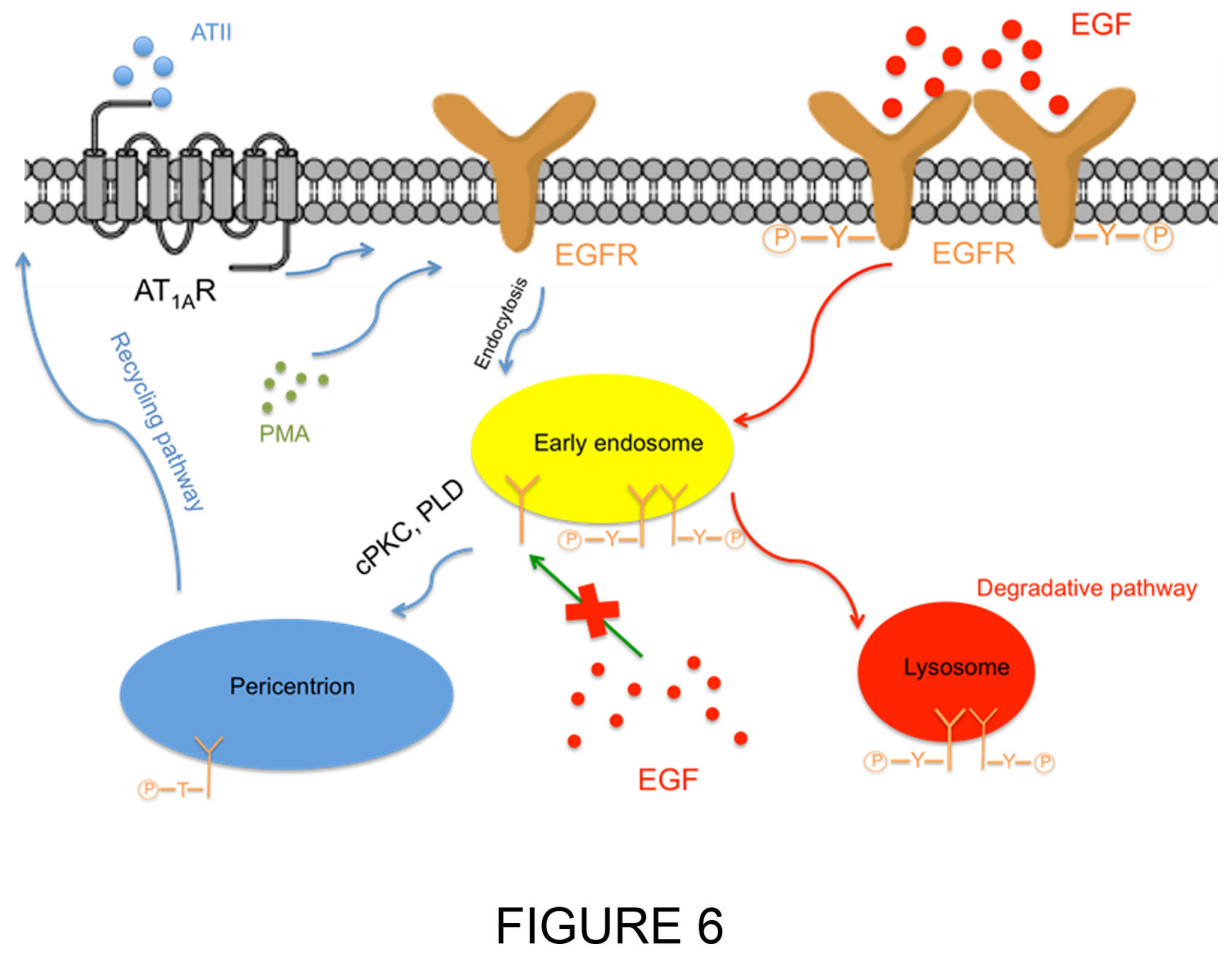
Scheme illustrating sustained activation of PKC induces PLD- and endocytosis- dependent phosphorylation of Thr-654 on EGFR and sequestration of EGFR to a cPKC- dependent subset of recycling compartment (pericentrion). Prolonged treatment with ATII or PMA could induce translocation of EGFR to pericentrion and phosphorylation of EGFR on Thr-654 on a cPKC- and PLD- dependent manner. Sequestration of EGFR to pericentrion protects EGFR accessed by EGF.

 The above results suggest that sequestration, Thr-654 phosphorylation, and protection of EGFR induced by AT-II are all pericentrion-dependent. Therefore, it became important to determine the mechanistic interactions of these effects and, more specifically, if phosphorylation of Thr-654 is essential for either protection or sequestration, and if so, then what is the mechanistic order of these events. To investigate this, a non-phosphorylatable EGFR-T654A mutant (TA-EGFR-GFP) was generated, and the effects of PMA on trafficking of this mutant were compared to wild-type EGFR (WT-EGFR-GP). As seen above, PMA induced translocation of WT-EGFR-GFP to the perinuclear region; strikingly, this was also observed for the mutant TA-EGFR-GFP to the perinuclear region (Figure 5A), demonstrating that Thr-654 phosphorylation is not required for sequestration, and also consistent with the results suggesting that sequestration of EGFR to the pericentrion is necessary for phosphorylation. Importantly, EGF was able to induce loss of TA-EGFR as with WT-EGFR, and with pretreatment of PMA EGF was able to induce the loss of TA-EGFR mutant (Figure 5B). This confirms that Thr-654 phosphorylation is not necessary for its translocation to the pericentrion but is essential for the protection. Thus, the formation of the pericentrion is required for phosphorylation on T654, which in turn is required for the protection of EGFR from accessed by EGF (Figure 6). 

## Discussion

 The pericentrion is a dynamic subset of recycling endosomes formed upon sustained stimulation with PMA or GPCR ligands, and requiring both PKC and PLD activities. However, to date, the function of the pericentrion in GPCR signaling has remained unclear. Here, we have established a novel role for the pericentrion in regulating EGFR phosphorylation, its intracellular trafficking and cellular fate. This could be of particular relevance in pathologies where cPKC and/or PLD activity are increased.

 To date, a number of studies have implicated PKC in the regulation of EGFR including its transactivation [28,29] and degradation [30-32]. Despite this, the mechanisms underlying these processes have remained unclear. The results presented herein now establish a crucial role for the pericentrion in sequestration of the EGFR from access by EGF. These conclusions come from several lines of evidence. Firstly, sustained activation of PKC by AT-II inhibited the loss of EGFR induced by EGF treatment coincident with co-sequestration of EGFR and the AT_1A_R in the pericentrion. Importantly, both cPKC and PLD activities were required for both EGFR protection and sequestration, consistent with our previous studies characterizing the pericentrion as cPKC- and PLD-dependent [6,8] Moreover, disruption of clathrin-mediated endocytosis, previously shown to disrupt the pericentrion, also abrogated the protective effects of AT-II and PMA on EGFR. Finally, sustained PKC activation with PMA was sufficient to induce EGFR sequestration to the pericentrion and reduced EGF access, also in a cPKC and PLD-dependent manner. These results are also highly consistent with and build upon our previous study demonstrating co-sequestration of both the 5-HT receptor and the EGFR in the pericentrion following 5-HT stimulation [7]. By extending these previous findings, it is evident that both translocation of GPCRs to the pericentrion and the heterologous sequestration of other receptors are emerging as more generalized roles for sustained activation of cPKCs.

 Previous research has reported that phosphorylation of EGFR at various residues is important for regulating its trafficking. Indeed, PMA-induced phosphorylation of EGFR on Thr-654 was reported to change its fate from the degradative pathway to the recycling endosome [18]. An important conclusion from the current study emanates from the observation that phosphorylation of EGFR on Thr-654 shows delayed kinetics, and the results implicate the formation of the pericentrion in regulating EGFR phosphorylation by PKC. Thus, EGFR phosphorylation on Thr-654 was prevented by inhibiton of cPKC, PLD and endocytosis. These results place EGFR in a newly appreciated subset of PKC substrates that are phosphorylated with delayed kinetics [10]. However, this is clearly residue specific as the pericentrion was not required for effects of AT-II or PMA on Tyr-1045 or Tyr-1068. Indeed, results here place phosphorylation of both these residues as upstream of the pericentrion suggesting they likely are not important for the observed protective effect. In agreement with this, our results demonstrate that phosphorylation of Thr-654 is necessary for the EGFR protection – as evidenced by the loss of protection of the TA-EGFR mutant compared to WT-EGFR. However, in contrast to this, the TA-EGFR mutant was able to translocate to the pericentrion equally as well at WT-EGFR. This suggests that phosphorylation of Thr-654 is not a prerequisite for entry into the recycling endosomes but may be crucial for sequestering EGFR in the slow recycling pathway. These data also disclose a sequence of events whereby the EGFR is first translocated to the pericentrion, is phosphorylated on Thr-654, and is then protected from degradation by reducing the access of EGF to the EGFR (Figure 6). 

 There are several implications from these results, specifically when considering EGFR related mechanisms of oncogenesis and tumor biology, or pathologies wherein PKC and PLD activities are increased. For example, studies in breast cancer cell lines have reported that EGFR escapes from the degradative pathway to the recycling compartment, and that this contributes to their enhanced malignant phenotype [33,34]. Moreover, separate studies of breast cancer cell lines have found overexpression of cPKCs and implicated them in cell growth and proliferation [35,36]. Additionally, enhanced PLD activity in breast cancer is reported to correlate with increased invasion, migration and proliferation [37-40]. These studies suggest the intriguing possibility that, in these breast cancers, highly activated cPKCs induce the formation of the pericentrion, which facilitates the proliferation and migration of the cancer cells by sequestering and protecting EGFR from accessed by EGF. This could also be true for some non-small cell lung cancers in which cPKCs are highly expressed and the downregulation of EGFR is impaired [41-43]. These possibilities are currently undergoing further study in our laboratory.

 In conclusion, the results reveal a novel role for the pericentrion in regulating EGFR phosphorylation, intracellular trafficking, and fate by protecting EGFR from accessed by EGF. Phosphorylation of EGFR on Thr-654 was pericentrion- dependent and was required for the protection of EGFR. In many cancers, EGFR evades degradation by entering the recycling pathway, and this invites a role for PKC and the pericentrion in these EGF-induced oncogenic properties. 
